# An Isoform of Nedd4-2 Plays a Pivotal Role in Electrophysiological Cardiac Abnormalities

**DOI:** 10.3390/ijms18061268

**Published:** 2017-06-14

**Authors:** Shintaro Minegishi, Tomoaki Ishigami, Hisho Kawamura, Tabito Kino, Lin Chen, Rie Nakashima-Sasaki, Hiroshi Doi, Kengo Azushima, Hiromichi Wakui, Yumi Chiba, Kouichi Tamura

**Affiliations:** 1 Department of Medical Science and Cardiorenal Medicine, Graduate School of Medicine, Yokohama City University, Yokohama 236-0004, Japan; minegish@yokohama-cu.ac.jp (S.M.); e133027g@yokohama-cu.ac.jp (H.K.); kino-tabito@umin.ac.jp (T.K.); mysterylin@foxmail.com (L.C.); musika0720@yahoo.co.jp (R.N.-S.); t156052b@yokohama-cu.ac.jp (H.D.); azushima@yokohama-cu.ac.jp (K.A.); hiro1234@yokohama-cu.ac.jp (H.W.); tamukou@yokohama-cu.ac.jp (K.T.); 2 Department of Nursing, Graduate School of Medicine, Yokohama City University, Yokohama 236-0004, Japan; ychiba@yokohama-cu.ac.jp

**Keywords:** Nedd4-2, cardiac ion channels, salt sensitivity, hypertension, cardio-renal syndrome, cardiovascular disease

## Abstract

We have previously shown that neural precursor cell-expressed developmentally downregulated gene 4-2 (Nedd4-2) isoforms with a C2 domain are closely related to ubiquitination of epithelial sodium channel (ENaC), resulting in salt-sensitive hypertension by Nedd4-2 C2 targeting in mice. The sodium voltage-gated channel alpha subunit 5 (*SCN5A*) gene encodes the α subunit of the human cardiac voltage-gated sodium channel (I Na), and the potassium voltage-gated channel subfamily H member 2 (*KCNH2*) gene encodes rapidly activating delayed rectifier K channels (I Kr). Both ion channels have also been shown to bind to Nedd4-2 via a conserved Proline-Tyrosine (PY) motif in C-terminal with subsequent ubiquitination and degradation by proteasome. Therefore, loss of Nedd4-2 C2 isoform might be involved in electrophysiological impairment under various conditions. We demonstrate here that Nedd4-2 C2 isoform causes cardiac conduction change in resting condition as well as proarrhythmic change after acute myocardial infarction (MI). The Nedd4-2 C2 knockout (KO) mice showed bradycardia, prolonged QRS, QT intervals, and suppressed PR interval in resting condition. In addition, enhancement of T peak/T end interval was found in mice with surgical ligation of the distal left coronary artery. Morphological analyses based on both ultrasonography of the living heart, as well as histopathological findings revealed that Nedd4-2 C2 KO mice show no significant structural changes from wild-type littermates under resting conditions. These results suggested that Nedd4-2 with C2 domain might play an important role in cardio-renal syndrome through post-transcriptional modification of both ENaC and cardiac ion channels, which are critical for kidney and heart functions.

## 1. Introduction

Cardiovascular disorders (CVD) including ischemic heart diseases, congestive heart failures, and arrhythmic disorders, such as atrial fibrillation and ventricular arrhythmias, are becoming a global health burden, which could lead to major obstacles for fulfillment of human healthy longevity. Subjects with chronic kidney disease (CKD) have higher morbidity and mortality resulting from CVDs, suggesting that CKD and CVD have mutually intervening pathophysiological relations referred to as “cardio-renal associations” or “cardio-renal syndrome (CRS)” [1,2]. Detailed molecular pathophysiological dissections underlying both the kidney and heart should provide biological insight into cardio-renal syndrome and translational procedures, which lead to the prevention of serious cardiovascular complications associated with CKD.

Besides mutations directly affecting ion channels, post-transcriptic abnormalities of cell surface expressions and retrieval of these membranous transporters are responsible for hereditary salt-sensitive hypertension, such as Liddle syndrome [3]. Impaired intracellular trafficking for proteosomal degradations of epithelial sodium channel (ENaC) leads to persistent tubular surface expression of ENaC and enhanced salt reabsorption in terminal nephron. Mutations in the Proline-Tyrosine (PY)motif in C terminal of proteins abolished binding with Tryptophan-Tryptophan W domain of neural precursor cell-expressed developmentally downregulated gene 4-2 (Nedd4-2/L) and subsequent ubiquitination. Genes encoding for human and rodent Nedd4-2/L have molecular diversity with two to three isoform formations [4,5], one of which is critically regulated by functional common variant, rs4149601 [4,6,7], in humans. Our detailed analyses of transcriptional multiplicity revealed Nedd4-2/L with C2 domain isoform expressed restrictedly along distal tubules with ENaC [5,8]. Recently, we reported that Nedd4-2 C2 knockout (KO) mice showed salt-sensitive hypertension with suppressed salt excretion from tubules while receiving a high intake of oral salt and prolonged ENaC expression with disturbed ubiquitination for subsequent proteasomal degradation [9]. We demonstrated that loss of Nedd4-2 C2 isoform causes salt-sensitive hypertension only under conditions of a high dietary salt intake in vivo. The KO mice had reduced urinary sodium excretion and osmotic pressure and increased water intake and urine volume while receiving a high-salt diet. In contrast, there was no difference in metabolic data between wild-type and KO mice receiving a normal control diet.

Salt sensitivity and hypertension are common disorders and one of major contributing to the development of CKD, because the kidneys and heart are critical target organs in patients with salt sensitivity and hypertension. Ion channels are highly characteristic molecules of both the kidney and heart for maintaining physiological functions. In addition, both sodium voltage-gated channel alpha subunit 5 (SCN5A), voltage-gated sodium channels and potassium voltage-gated channel subfamily H member 2 (KCNH2), rapidly activating delayed rectifier K channels, which are critical for cardiac depolarization and repolarization [10], have PY-motif in their C-terminal that Nedd4-2 interact with [11,12,13,14]. Impaired ubiquitination by abolished Nedd4-2 function might be involved in electrophysiological disturbances that cause cardiac arrhythmic disorders. Accordingly, we hypothesized that gene-targeting of Nedd4-2 C2 should result in disturbed cardiac electrophysiological function, which might be exacerbated by disease conditions such as myocardial infarction. Therefore, we performed the present experiments, including both electrophysiological and structural examinations, in Nedd4-2 C2 KO mice in resting condition and artificially-generated myocardial infarction with the use of sophisticated micro-surgical techniques. The Nedd4-2 C2 KO mice showed bradycardia, prolonged QRS, QTc intervals, and suppressed PR intervals in resting condition. In addition to prolonged QTc interval, enhancement of T peak/T end interval was found in mice with surgical ligation of the distal left coronary artery. Morphological analyses based on both ultrasonographic examination of the living heart and histopathological findings revealed that Nedd4-2 C2 KO mice show no significant structural changes from their wild-type littermates. These results suggested that Nedd4-2 with C2 domain might play a pivotal role in cardio-renal associations through post-transcriptional modification of both ENaC and cardiac ion channels, which are critical for kidney and heart functions.

## 2. Results

### 2.1. Cardiac Phenotypic Evaluations in Neural Precursor Cell-Expressed Developmentally Downregulated Gene 4-2 (Nedd4-2) C2 Knock out (KO) Mice

#### 2.1.1. Comparison of Electrocardiographic and Echocardiographic Findings between Wild-Type and Nedd4-2 C2 Knockout Mice under Resting Conditions

**Figure 1:**
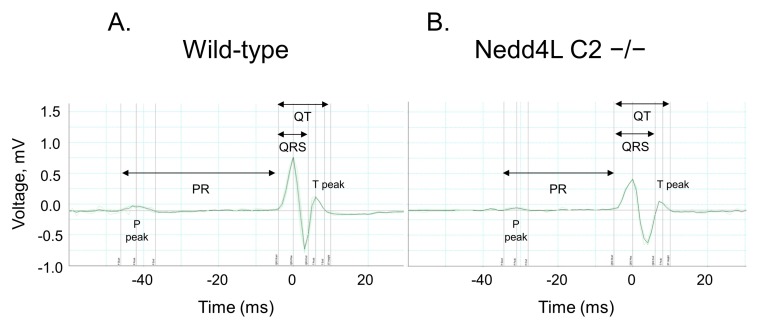
Representative surface electrocardiogram (ECG) traces recorded in wild-type and neural precursor cell-expressed developmentally downregulated gene 4-2 (Nedd4-2) C2-knockout mice. Representative ECG traces from wild-type (**A**) and Nedd4-2 C2-knockout mice (**B**).

To characterize electrophysiological features, we performed surface electrocardiography (ECG) analysis using male Nedd4-2 C2 KO mice (*n* = 5; 8 weeks old) and their wild-type littermates (*n* = 5; 8–9 weeks old) under resting conditions. The various ECG parameters were measured and analyzed. Representative ECG tracings are shown in Figure 1, and mean ECG parameters are shown in Table 1. The PR interval of Nedd4-2 C2 KO mice was significantly shorter than that of wild-type mice (*p* = 0.0104). The P-wave duration was significantly increased in Nedd4-2 C2 KO mice (*p* < 0.0001). In addition, prolonged QT interval and QTc were found in Nedd4-2 C2 KO mice (*p* < 0.0001). Furthermore, we performed transthoracic echocardiography (UCG) analysis using the same mice under resting conditions. The UCG parameters are shown in Table 2. There was no difference in UCG findings between Nedd4-2 C2 KO mice and wild-type mice.

#### 2.1.2. Comparison of Heart Rate and Heart Rate Variability between Wild-Type and Nedd4-2 C2 Knockout Mice in Resting Conditions

**Figure 2:**
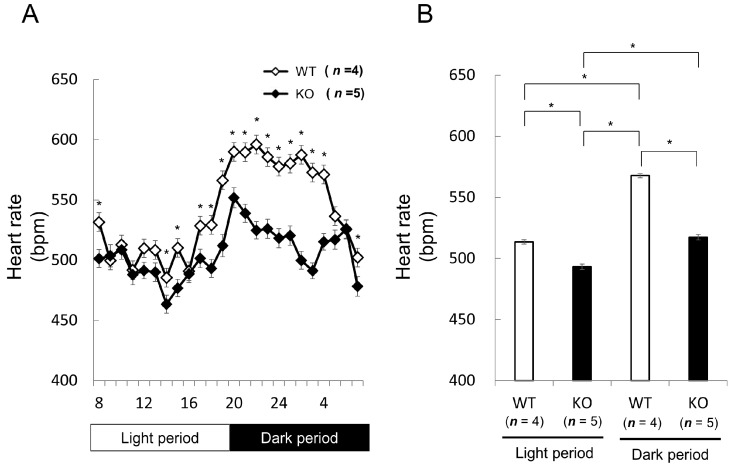

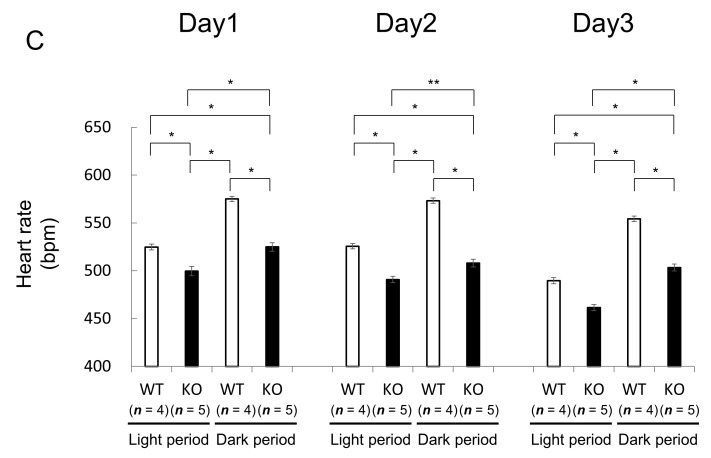
Comparison of basal heart rate between wild-type (WT) and Nedd4-2 C2-knockout (KO) mice assessed by radiotelemetry. Male wild-type littermates (*n*  =  4; 14–16 weeks old) and Nedd4-2 C2 −/− mice (*n*  =  5; 14–16 weeks old) were used for this experiment. Telemetered, long-term ECG recordings showed that Nedd4-2 C2 knock-out mice had significantly slower mean heart rates compared with wild-type mice during both the dark and light periods. (**A**) * *p* < 0.05, (**B**) * *p* < 0.0001. Statistical analyses were performed by repeated measures analysis of variance (ANOVA) followed by Bonferroni multiple comparison tests. * *p* < 0.0001, ** *p* = 0.0001 (**C**).

**Figure 3:**
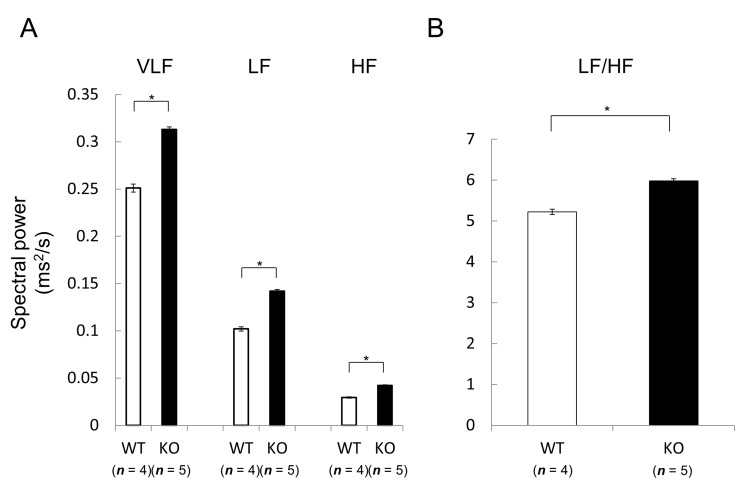
Comparison of basal heart rate variability between wild-type and Nedd4-2 C2-knockout mice assessed by radiotelemetry. Male wild-type littermates (*n*  =  4; 14–16 weeks old) and Nedd4-2 C2 −/− mice (*n*  =  5; 14–16 weeks old) were used for this experiment. VLF, LF and HF components of heart rate variability were significantly higher in Nedd4-2 C2 knock out (KO) knockout mice than wild-type mice (**A**). The coefficient between LF and HF spectral power density, which is presumed to reflect sympatho-vagal balance, was significantly increased in Nedd4-2 C2 KO mice (**B**). VLF; Very low frequency, HF; High-frequency, LF; Low frequency. * *p* < 0.0001.

We next studied heart rates and rhythm in Nedd4-2 C2 KO mice. The opposing effects of sympathetic and parasympathetic activity generate beat-to-beat fluctuations in heart rate [15,16]. Heart rate variability (HRV) reflects the balance between the sympathetic and parasympathetic nervous systems. Mean heart rate and HRV were measured by radiotelemetry in male wild-type littermates (*n* = 2; 14–16 weeks old) and Nedd4-2 C2 KO mice (*n* = 2; 14–16 weeks old). Resting mean heart rate was significantly slower in Nedd4-2 C2 knock-out mice during both the dark and light periods (Figure 2). Interestingly, the low frequency/high frequency (LF/HF) ratio, indicating the relative strength of the sympatho-vagal balance, was significantly higher in Nedd4-2 C2 KO mice than in wild-type (*p* < 0.0001; Figure 3).

#### 2.1.3. Comparison of Electrocardiographic and Echocardiographic Findings between Wild-Type and Nedd4-2 C2 Knockout Mice after Myocardial Infarction

**Figure 4:**
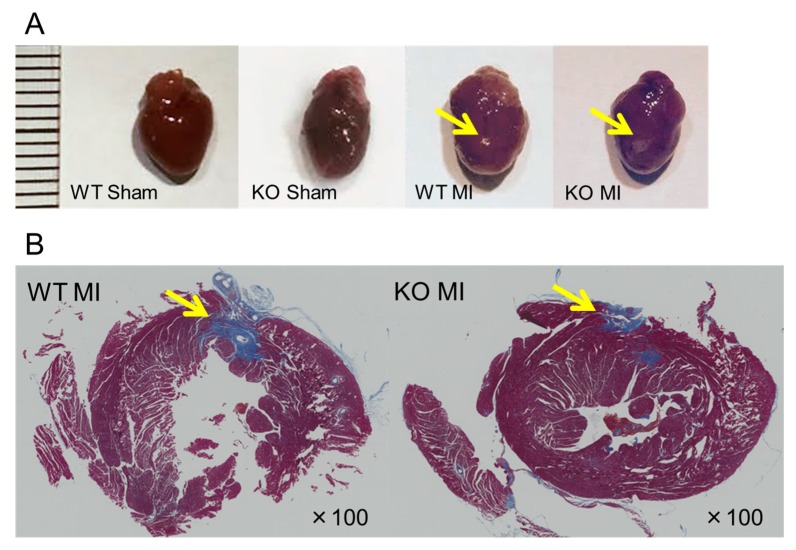
Representative hearts from sham-operated and left anterior descending artery ligated mice. Whole hearts (**A**) and microscopic findings (**B**) of the infarcted regions are shown (arrows). Sections stained with Masson’s trichrome stain (**B**) are shown by ×100 magnification under light microscopic examination using a BZ-9000 fluorescence microscope (Keyence, Osaka, Japan). The ruler shows millimeter markings. WT: wild-type, KO: knockout, MI: myocardial infarction.

**Figure 5:**
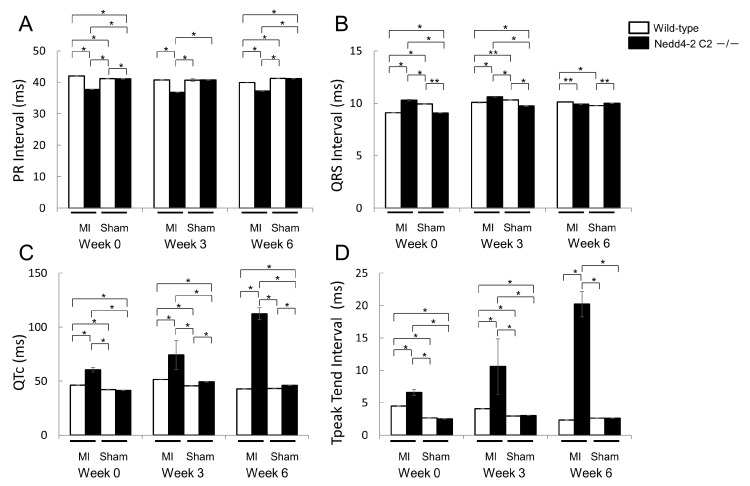
Comparison of electrocardiographic findings between wild-type and Nedd4-2 C2-knockout mice after myocardial infarction. Male wild-type littermates (*n* = 4; 11–14 weeks old) and Nedd4-2 C2 −/− mice (*n* = 4; 11–14 weeks old) were used for this experiment. A for PR intervals, B for QRS intervals, C for QTc intervals, and D for T peak T end intervals, respectively. Data are expressed as means ± SE. Nedd4-2 C2-knockout mice showed enhancement of T peak/T end interval with surgical ligation of the distal left coronary artery. MI; myocardial infarction. * *p* < 0.0001, ** *p* < 0.001.

To further evaluate cardiac function, we performed ECG and UCG analyses after ligation of the distal LAD (left anterior descending artery). Age-matched (11–14 week-old) male Nedd4-2 C2 KO mice and wild-type littermates were divided into four groups as follows: (1) wild-type underwent sham-operated surgery (wild-type sham group, *n* = 4); (2) wild-type underwent MI surgery (wild-type MI group, *n* = 4); (3) KO underwent sham-operated surgery (KO sham group, *n* = 4); and (4) KO underwent MI surgery (KO MI group, *n* = 4). Figure 4 shows representative hearts of the sham-operated and MI mice. Masson’s trichrome staining revealed a similar degree of infarct size in the wild-type and Nedd4-2 C2 KO mice. During 6 weeks after MI, PR interval of Nedd4-2 C2 KO MI mice was significantly shorter than that of wild-type MI mice (*p* < 0.0001; Figure 5). Prolonged QT interval and QTc were found in Nedd4-2 C2 KO MI mice (*p* < 0.0001; Figure 5). In addition to prolonged QTc interval, enhancement of T peak/T end interval was found in Nedd4-2 C2 KO MI mice (*p* < 0.0001; Figure 5). In contrast, there was no difference in UCG findings between KO MI mice and wild-type MI mice at week 6 (Table 3).

#### 2.1.4. Comparison of Blood Chemistry and Urinalysis between Wild-Type and Nedd4-2 C2 Knockout Mice under Resting Conditions

**Figure 6:**
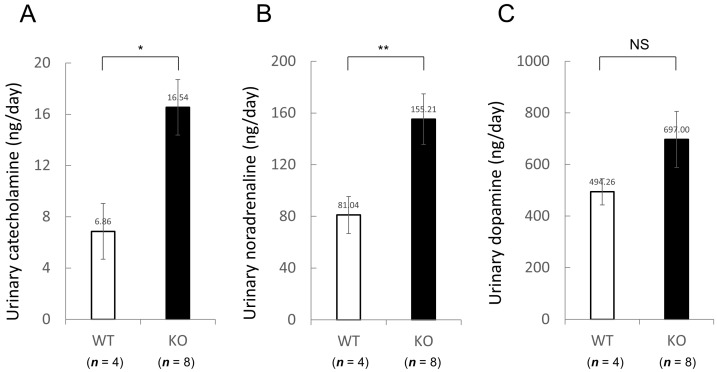
Increased daily urinary catecholamine excretion in Nedd4L C2-knockout (KO) mice. Daily urinary (**A**) catecholamine, (**B**) noradrenaline, and (**C**) dopamine excretion in wild-type (WT) and Nedd4L C2 KO mice. Male wild-type littermates (*n* = 4; 8–9 weeks old) and Nedd4-2 C2 −/− mice (*n* = 8; 8 weeks old) were used for this experiment. Data are expressed as means ± SE. Statistical analyses were performed by unpaired, two-tailed Student *t* test. * *p* = 0.0191, ** *p* = 0.0329, NS; not significant.

Moreover, we evaluated serum creatinine (Cr), sodium (Na), calcium (Ca) and urinary catecholamine levels. Serum Cr, Na and Ca levels were also similar between two groups under resting conditions (Table 4). In comparison with wild-type mice, the daily urinary catecholamine excretion was significantly increased in Nedd4-2 C2 KO mice (Figure 6).

## 3. Discussion

Recently, it is proposed that impaired renal function mutually promotes impaired cardiac function, a phenomenon that is referred to as “cardio-renal associations” or “cardio-renal syndrome (CRS)”. Acute kidney injuries are frequently accompanied by congestive heart failure in clinical practice [17,18]. Worsening and improving cardiac functions are concurrently expected to result in worsening and improving renal function. Because renal blood flow consumes approximately 20–30% of cardiac output per unit, impaired hemodynamics of the glomerular circulation might be responsible for this relationship. In addition, various endocrinological factors, such as natriuretic peptides that are released from cardiomyocytes, and promote natriuresis by suppressing sodium reabsorption in tubules. Impaired production of erythropoietin, one of hematopoietic endocrinological factors mainly generated and secreted from the kidney, promotes renal anemia and aggravates cardiac functions. Therefore, these cardio-vascular-endocrinological factors are possibly potent descriptive factors for this relationship. However, whether molecular components commonly expressed in both kidney and heart participate in this relationship remains unclear.

Both cardiac ion channels and tubular sodium channels are functionally critical molecules for the basal physiological properties of the kidney and heart. Electrically-synchronized cardiac contraction and relaxation cycles are essential for maintaining homeostatic blood circulation. Dysrhythmic disorders such as atrial fibrillation, ventricular tachycardia, and extrasystoles cause either lethal or non-lethal cardiac events, especially under diseased conditions such as myocardial infarction. Hypertension and salt sensitivity are major risk factors for the development of CKD (chronic kidney disease). Molecular genetic analyses of hereditary familial hypertension have disclosed that impaired sodium re-absorption in tubules by sodium transporters and their accessory proteins are responsible for salt sensitivity and hypertension [19,20]. In addition, congenital abnormalities for genes directly involved in cardiac ion channels cause both tachy- and brady-hereditary arrhythmic disorders such as hereditary long and short QT syndromes [21,22,23,24,25], Brugada syndrome [26], hereditary sick sinus syndrome [27], and congenital atrioventricular block [28].

Currently, we are performing phenotypic analyses of the Nedd4-2 C2 KO mice using electrocardiography, telemetry measurement of heart rate and heart rate variability, and ultrasonography during a normal diet with or without distal coronary artery ligation. In resting conditions, the Nedd4-2 C2 KO mice showed significant bradycardia, prolonged QRS, QT intervals, and suppressed PR interval in spite of enhancement of catecholamine secretion in urine, suggesting impaired depolarization and repolarization in the cardiac conduction system. (Figure 3 and Figure 6) Interestingly, the low frequency/high frequency (LF/HF) ratio, indicating the relative strength of the sympatho-vagal balance, was significantly higher in Nedd4-2 C2 KO mice than in wild-type mice. The opposing effects of sympathetic and parasympathetic activity generate beat-to-beat fluctuations in heart rate. HRV reflects the balance between the sympathetic and parasympathetic nervous systems [15,16]. To further evaluate cardiac function, we performed ECG and UCG analyses after ligation of the distal coronary artery. During 6 weeks after MI, PR interval of Nedd4-2 C2 KO MI mice was significantly shorter than that of wild-type MI mice. Prolonged QT interval and QTc were found in Nedd4-2 C2 KO MI mice. In addition, enhancement of T peak/T end interval were found in Nedd4-2 C2 KO MI mice. These suggested that abnormalities in the repolarization process in the cardiac conduction system were present in Nedd4-2 C2 KO mice under diseased conditions. There was no difference in UCG findings between KO MI mice and wild-type MI mice at week 3 and 6 despite the earlier reduction of CO and SV in Nedd4-2 C2 KO MI mice (Table 3).

As mentioned above, impaired post-transcriptional modification of ENaC by Nedd4-2/L caused hereditary salt sensitive hypertension in vivo, and genetic abnormalities of cardiac ion channels directly led to serious hereditary arrhythmic disorders. Whether impaired post-transcriptional modifications of cardiac ion channels cause ECG abnormalities has not been clearly demonstrated yet. Both SCN5A [11] and KCNH2 [13,14], which are critically involved in cardiac depolarization/repolarization process, were found to have PY motifs in their C-terminal and have post-transcriptional regulation by Nedd4-2/L in vitro. To determine whether impaired ubiquitination for cardiac ion channels causes ECG abnormalities, we performed detailed phenotypic analyses of originally generated Nedd4-2 C2 KO mice. Our detailed electrophysiological and ultrasonographic in vivo analyses of Nedd4-2 C2 KO mice revealed that loss of Nedd4-2 C2 isoform promotes electrophysiological abnormalities. Not only under resting conditions but also under the diseased condition of myocardial infarction, mice without Nedd4-2 C2 showed progressively prolonged QTc intervals and enhanced T peak/T end interval, which might have lethal proarrythmic properties with myocardial infarction, resulting in ventricular tachy-arrythmia and cardiac sudden death. Because our current analyses were performed during a normal diet throughout the experiments, blood pressure elevation was unlikely to influence organ damage. Therefore, these cardiac phenotypic changes were independent of the target organ damage caused by salt sensitivity and hypertension. Melander et al. reported that subjects with rs4149601 which we reported previously [4] showed low-renin, salt-sensitive hypertension with significantly increased cardiovascular mortality in a prospective clinical observational study [29,30]. Our current experiments support the notion that suppressed Nedd4-2/Nedd4L function causes significant cardiac arrhythmic involvement through post-transcriptional modification of cardiac ion-channels. Subjects with salt sensitivity and hypertension due to Nedd4L abnormalities might be at higher risk for more serious cardiovascular involvement than those without Nedd4L abnormalities.

In summary, we demonstrated that Nedd4-2 C2 KO mice, which showed salt sensitivity and hypertension during a high oral-salt intake, have electrocardiographic abnormalities without structural changes, possibly due to impaired post-transcriptional modifications of cardiac ion channels. However our current analyses using originally developed genetically-engineered mice showed loss of Nedd4-2 C2 isoform cause significant electrophysiological change of heart, detailed molecular insight remains unclear. Further studies such as immunohistochemistry for a certain cardiac conduction system or patch-clamp test for cardiomyocyte may help to elucidate detailed molecular mechanisms underlying these phenotypes. Finally, Nedd4-2 C2 isoform expressed in both kidney and heart plays a pivotal role in post-transcriptional modifications of sodium ion transporter and cardiac ion channels. Therefore, Nedd4-2 might act as molecules converging the pathophysiological basis of cardio-renal associations.

## 4. Materials and Methods

### 4.1. Generation of Nedd4-2 C2 Domain Knockout Mice

Using genomic, cDNA, and expressed sequence tag (EST) databases, including GenBank nr and Mouse EST entries, we initially performed BLAST and cross match analyses. Then, the results of analyses were parsed with chromosome 18 to form a consistent assembly of EST, cDNA, and genomic sequence. We synthesized targeting vector designed to disrupt newly discovered exon 2 coding Nedd4-2 C2 domain, and subsequently generated Nedd4-2 C2 −/− mice. The detailed technical strategy for Nedd4-2 C2 knockout (KO) mice is described in our previous experiments [9]. All animal experiments were performed in accordance with the guidelines of the Animal Experiment Committee, Yokohama City University School of Medicine, and with approval of the Animal Experiment Committee, Yokohama City University School of Medicine (F-A-14-088, 31 March 2014).

### 4.2. Electrocardiographic Analyses

Prior to examination, mice were anesthetized by inhalation of 4% isoflurane in a glass chamber. Subsequently, anesthesia was maintained during procedures by 1% to 1.5% isoflurane, administered via nose cones. Electrocardiography (ECG) was performed using a PowerLab system (AD Instruments, Sydney, Australia). ECG signal processing was performed with the software program LabChart (Sydney, Australia). The following parameters were investigated: PR interval, P-wave duration, QTc, QRS, and QT intervals.

### 4.3. Echocardiographic Analyses

Mice were anesthetized by inhalation of 4% isoflurane in a glass chamber. Anesthesia was then maintained during procedures by 1% to 1.5% isoflurane, administered via nose cones. Transthoracic echocardiography (UCG) was performed using a Aplio SSA-700A echocardiograph (Toshiba, Tokyo, Japan). The two-dimensional and M-mode images were obtained in the parasternal short and long axis view of the left ventricle. Using the M-mode image, individual parameters were measured as follows: left ventricle internal diameter in diastole and systole (LVIDd and LVIDs), end diastolic volume, end systolic volume and stroke volumes (EDV, ESV, and SV), cardiac output (CO), fractional shortening (FS), ejection fraction (EF), intraventricular septal thickness, and left ventricular posterior wall thickness in diastole (IVSTd and LVPWd) and systole (IVSTs and LVPWs).

### 4.4. Heart Rate and Heart Rate Variability Measurements

Heart rate and heart rate variability (HRV) was measured by radiotelemetry (HD-X11, ETA-F10, DYWIDAG-Systems International, Bolingbrook, IL, USA), as described previously [9]. Mice were anesthetized, and a pressure-sensing catheter was implanted into the left carotid artery (HD-X11) or subcutaneously (ETA-F10). After the surgical recovery period, each mouse was housed individually under a 12-h light-dark cycle. Heart rate were recorded every minute by the device. HRV was quantified with the HRV plug-in for Chart v5.0 (AD Instruments, Sydney, Australia).

### 4.5. Examinations for Left Anterior Descending Artery Ligation Model

To further evaluate the electrophysiological and morphological functions of Nedd4-2 C2 KO mice, we used a small myocardial infarction (MI) model prepared by visually confirmed ligation of the distal left anterior descending artery (LAD) using microsurgery. Mice were anesthetized with 4% isoflurane, and anesthesia was then maintained during procedures with 1% to 1.5% isoflurane and oxygen. The mice were mechanically ventilated after intubation. The heart was exposed at the 4th or 5th intercostal space. The left anterior descending coronary artery was ligated 3 mm from the left auricle with 8–0 silk suture, and the thoracic wall was closed with 5–0 nylon suture. Sham operations were identical, except for the ligation of the LAD. Cardiac function was monitored by ECG and UCG on day 1, week 3, and week 6 after surgery. MI sizes were qualitatively evaluated by Masson’s staining.

### 4.6. Catecholamine Determination

Catecholamine levels were determined from urine samples collected over a 24-h period using metabolic cages (SN-781, Shinano Manufacturing Co., Ltd., Tokyo, Japan). Urine samples were stored at −80 °C until testing. Adrenaline, and dopamine concentrations were quantified by high-performance liquid chromatography (HPLC).

### 4.7. Blood Chemistry Analysis

Blood was collected by cardiac puncture, and all samples were allowed to coagulate for several hours before separation. After centrifugation at 1200× *g* for 20 min at 4 °C, serum was separated and stored at −80 °C until testing. Creatinine (Cr), sodium (Na) and calcium (Ca) levels were measured according to the manufacturer’s instructions (Oriental Yeast Co., Ltd., Tokyo, Japan).

### 4.8. Statistical Analyses

Data are expressed as means ± SE. Statistical analyses were performed with SPSS software (Dr. SPSS, SPSS Japan, Tokyo, Japan) and StatView (ver. 5, SAS, Cary, NC, USA). Repeated measured ANOVA and subsequent post-hoc analyses (Bonferroni) were used for Figure 2A,B and Figure 5 and Table 3. Unpaired, two-tailed Student *t* test were used for Figure 3A,B and Figure 6 and Table 1, Table 2, and Table 4. *p* < 0.05 was considered to indicate statistical significance.

## Figures and Tables

**Table 1:** Comparison of electrocardiographic findings between wild-type and neural precursor cell-expressed developmentally downregulated gene 4-2 (Nedd4-2) C2-knockout mice. Male wild-type littermates (*n* = 5; 8–9 weeks old) and Nedd4-2 C2 −/− mice (*n* = 5; 8 weeks old) were used in this experiment. Data are expressed as means ± standard errors (SE). Nedd4-2 C2-knockout mice showed a shorter PR interval than wild-type mice. P-wave duration in Nedd4-2 C2-knockout mice was longer than that in the wild-type mice. Moreover, prolonged QRS, QT and QTc were found in Nedd4-2 C2-knockout mice.

ECG Waveform	Wild-Type	Nedd4-2 C2 −/−	*p*
	(*n* = 5)	(*n* = 5)	
PR Interval (ms)	40.81 ± 0.10	40.38 ± 0.12	0.0104
P Duration (ms)	15.00 ± 0.34	17.46 ± 0.28	<0.0001
QRS Interval (ms)	8.79 ± 0.02	9.97 ± 0.04	<0.0001
QT Interval (ms)	13.34 ± 0.03	18.18 ± 0.44	<0.0001
QTc (ms)	38.97 ± 0.08	53.52 ± 1.27	<0.0001

**Table 2:** Comparison of echocardiographic (UCG) findings between wild-type and Nedd4-2 C2-knockout mice. Male wild-type littermates (*n* = 5; 8–9 weeks old) and Nedd4-2 C2 −/− mice (*n* = 5; 8 weeks old) were used in this experiment. Data are expressed as means ± SE. Echocardiographic parameters showed no difference between wild-type and Nedd4-2 C2-knockout mice. IVSTd, intraventricular septal thickness in diastole; LVIDd, left ventricle internal diameter in diastole; LVPWTd, left ventricular posterior wall thickness in diastole; IVSTs, intraventricular septal thickness in systole; LVIDs, left ventricle internal diameter in systole; LVPWTs, left ventricular posterior wall thickness in systole; EDV, end diastolic volume; ESV, end systolic volume; SV, stroke volume; CO, cardiac output; EF, ejection fraction; FS, fractional shortening; HR, heart rate.

UCG Measurements	Wild-Type	Nedd4-2 C2 −/−	*p*
	(*n* = 5)	(*n* = 5)	
IVSTd (mm)	0.82 ± 0.02	0.81 ± 0.02	0.7717
LVIDd (mm)	4.28 ± 0.07	4.29 ± 0.04	0.9484
LVPWTd (mm)	0.73 ± 0.02	0.75 ± 0.02	0.3991
IVSTs (mm)	1.21 ± 0.03	1.15 ± 0.04	0.2290
LVIDs (mm)	3.00 ± 0.08	3.00 ± 0.05	0.9781
LVPWTs (mm)	0.92 ± 0.02	0.97 ± 0.02	0.0961
EDV (μL)	80.14 ± 3.99	79.08 ± 2.29	0.8291
ESV (μL)	28.76 ± 2.35	27.58 ± 1.28	0.6799
SV (μL)	51.38 ± 2.28	51.50 ± 1.69	0.9675
CO (mL/min)	24.95 ± 1.12	24.44 ± 0.89	0.7310
EF (%)	65.20 ± 1.68	65.21 ± 1.28	0.9955
FS (%)	30.16 ± 1.14	29.96 ± 0.86	0.8946
HR (bpm)	485.79 ± 5.33	473.46 ± 4.11	0.0819

**Table 3:** Comparison of echocardiographic findings between wild-type and Nedd4-2 C2-knockout mice after myocardial infarction. Male wild-type littermates (*n* = 4; 11–14 weeks old) and Nedd4-2 C2 −/− mice (*n* = 4; 11–14 weeks old) were used for this experiment. Data are expressed as means ± SE. MI, myocardial infarction; IVSTd, intraventricular septal thickness in diastole; SV, stroke volume; CO, cardiac output; EF, ejection fraction; FS, fractional shortening; ANOVA, two-way analysis of variance. ^a^ *p* < 0.05 vs. wild-type MI, wild-type sham, Nedd4-2 C2-knockout sham; ^b^ *p* < 0.05 vs. wild-type sham, Nedd4-2 C2-knockout sham.

UCG Measrements	Wild-Type	Nedd4-2 C2 −/−	Wild-Type	Nedd4-2 C2 −/−	*p*
	MI	MI	Sham	Sham	
	(*n* = 4)	(*n* = 4)	(*n* = 4)	(*n* = 4)	(ANOVA)
Week 0					
IVSTd (mm)	0.73 ± 0.02	0.72 ± 0.02	0.72 ± 0.02	0.75 ± 0.01	0.5464
SV (μL)	44.59 ± 3.69	31.67 ± 1.51 ^a^	45.80 ± 2.38	41.88 ± 1.18	0.0002
CO (mL/min)	22.81 ± 2.09	16.22 ± 0.75 ^a^	22.40 ± 1.28	20.97 ± 0.62	0.0017
EF (%)	63.57 ± 1.84	67.99 ± 1.17	68.25 ± 1.52	66.67 ± 1.18	0.1427
FS (%)	29.00 ± 1.15	31.76 ± 0.81	32.16 ± 1.07	31.14 ± 0.87	0.1860
Week 3					
IVSTd (mm)	0.70 ± 0.03	0.69 ± 0.02 ^b^	0.76 ± 0.01	0.75 ± 0.01	0.0327
SV (μL)	57.07 ± 4.85	51.09 ± 1.87	50.67 ± 4.20	49.50 ± 1.77	0.3844
CO (mL/min)	27.42 ± 2.26	25.29 ± 0.90	24.44 ± 0.89	24.95 ± 1.12	0.3852
EF (%)	69.16 ± 1.23	68.81 ± 1.09	67.99 ± 3.13	68.17 ± 1.14	0.9708
FS (%)	32.60 ± 0.86	32.44 ± 0.76	33.68 ± 0.58	32.00 ± 0.80	0.4587
Week 6					
IVSTd (mm)	0.75 ± 0.02	0.74 ± 0.02	0.77 ± 0.02	0.77 ± 0.02	0.5427
SV (μL)	58.81 ± 5.38	48.68 ± 1.62	54.80 ± 2.38	58.17 ± 2.69	0.1224
CO (mL/min)	29.93 ± 2.86	24.33 ± 0.66	27.05 ± 1.22	28.37 ± 1.39	0.1350
EF (%)	69.12 ± 1.11	70.45 ± 0.89	70.00 ± 0.89	69.86 ± 0.99	0.8338
FS (%)	32.63 ± 0.77	33.39 ± 0.63	33.35 ± 0.66	33.20 ± 0.73	0.8751

**Table 4:** Serum markers in wild-type and Nedd4L C2-knockout mice. Male wild-type littermates (*n* = 8; 7–10 weeks old) and Nedd4-2 C2 −/− mice (*n* = 9; 7–9 weeks old) were used for this experiment. Data are expressed as means ± SE. Cr, creatinine; Na, sodium; Ca, calcium.

Serum Markers	Wild-Type	Nedd4L C2 −/−	*p*
	(*n* = 8)	(*n* = 9)	
Serum Cr (mg/dL)	0.16 ± 0.01	0.16 ± 0.01	0.6433
Serum Na (mEq/L)	147.88 ± 1.36	144.56 ± 1.07	0.0708
Serum Ca (mg/dL)	9.19 ± 0.25	8.50 ± 0.21	0.0506

## Abbreviations

CKD	Chronic kidney disease
CVD	Cardiovascular disease
HRV	Heart rate variability
MI	Myocardial infarction
LAD	Left anterior descending (artery)
